# Supplementary material for: Complexome profiling of the Chlamydomonas psb28 mutant reveals TEF5 as an early PSII assembly factor
Source: Plant Cell. 2025 Mar 17;37(6):koaf055. doi: 10.1093/plcell/koaf055 (PMC12164590; doi:10.1093/plcell/koaf055)
Supplement: koaf055_Supplementary_Data [file koaf055_supplementary_data.zip › psb28_interactive.zip!/plots/intensity/qualityPlots/peptides/PTOX1_Cre07.g350750.t1.2.html]

### Cre07.g350750.t1.2

Trivial name: PTOX1  
SeqCoverage [median]: 14.50  
Mass: 45.74 kDa  
  
 

| Sequence | MissedCleavage | Mass [Da] | wtIntensity | muIntensity | Score |
| --- | --- | --- | --- | --- | --- |
| ALEAAQVLEPLRPR | 1 | 1561.89 | 2424.92 | 17663.49 | 84.29 |
| AVTLDGVTR | 0 | 930.51 | 1010.36 | 2188.14 | 66.07 |
| FDDYSTISEANPPR | 0 | 1610.72 | 306.87 | 1164.68 | 155.97 |
| SQDLYMFDSFQTSQPMQNPR | 0 | 2419.06 | 0.00 | 1541.03 | 57.41 |
|  |  |  | 3742.2 | 22557.3 |  |
